# Management of cardiometabolic risk in prostate cancer: expert consensus from Singapore and Malaysia

**DOI:** 10.3389/fonc.2026.1781791

**Published:** 2026-10-21

**Authors:** Kenneth Chen, Edmund Chiong, Ravindran Kanesvaran, Christopher Chieh Yang Koo, Warren Hwa Loon Lo, Alexander Loch, Rohan Malek, Choon Ta Ng, Marniza Saad, Muthukkumaran Thiagarajan, Teng Aik Ong, Jeffrey Kit Loong Tuan

**Affiliations:** 1 Department of Urology, Singapore General Hospital, Singapore, Singapore; 2 Department of Urology, National University Hospital, Singapore, Singapore; 3 Department of Surgery, Yong Loo Lin School of Medicine, Singapore, Singapore; 4 Division of Medical Oncology, National Cancer Centre Singapore, Singapore, Singapore; 5 Department of Cardiology, National University Heart Centre, Singapore, Singapore; 6 Department of Urology, Hospital Kuala Lumpur, Kuala Lumpur, Malaysia; 7 Department of Medicine, Universiti Malaya, Kuala Lumpur, Malaysia; 8 Damansara Specialist Hospital 2, Kuala Lumpur, Malaysia; 9 Department of Cardiology, National Heart Centre Singapore, Singapore, Singapore; 10 Cardiovascular Medicine, SingHealth Duke-NUS Academic Medical Centre, Singapore, Singapore; 11 Department of Clinical Oncology, Universiti Malaya, Kuala Lumpur, Malaysia; 12 Department of Radiotherapy and Oncology, Hospital Kuala Lumpur, Kuala Lumpur, Malaysia; 13 Department of Surgery, Faculty of Medicine, Universiti Malaya, Kuala Lumpur, Malaysia; 14 Department of Gastrointestinal Hepato pancreato-biliary and Urology Radiation Oncology, National Cancer Centre Singapore, Singapore, Singapore; 15 Oncology Academic Clinical Program, Duke-NUS Medical School, Singapore, Singapore

**Keywords:** androgen deprivation therapy, androgen receptor-targeted therapy, cardiometabolic risk, consensus recommendations, prostate cancer

## Abstract

**Background:**

Prostate cancer is one of the most diagnosed malignancies among men globally, with androgen deprivation therapy (ADT) and androgen receptor-targeted therapy remaining central to the management of advanced disease. Despite their proven efficacy in controlling tumour progression, these therapies are associated with significant cardiovascular and metabolic risks. The authors aimed to develop a set of evidence-based recommendations for the assessment, monitoring, and management of cardiometabolic risks associated with ADT and androgen receptor-targeted therapy in patients with prostate cancer.

**Methods:**

A panel of 12 clinicians from Malaysia and Singapore, with expertise in prostate cancer and cardiovascular disease management, developed consensus statements using a modified Delphi method. Statements were informed by a literature review and refined through structured discussions and anonymous voting until consensus was achieved.

**Results:**

A total of 13 statements were developed, of which 12 achieved consensus. Key recommendations include conducting a baseline evaluation of lifestyle risk factors and comorbidities, implementing a simplified cardiovascular disease risk assessment checklist, and tailoring treatment plans and structured management protocols according to individual cardiovascular disease risk categories. The panel also recommended referring high-risk or symptomatic patients for appropriate care (if not already on follow-up) and initiating cardiometabolic monitoring post-treatment with frequency adjusted based on patient-specific risk profiles. Additional endorsed strategies included incorporating lifestyle modification counselling during follow-up, applying the modified ABCDE Cardio-Oncology framework, and involving multidisciplinary teams in managing patients with elevated cardiovascular risk. The rejected statement, which proposed routine baseline electrocardiograms for all patients, lacked support owing to limited evidence, logistical and interpretative challenges, and concerns over delaying cancer treatment.

**Conclusions:**

This consensus document offers practical, evidence-based guidance for minimising cardiometabolic risks in patients with prostate cancer on ADT or androgen receptor-targeted therapy. These recommendations support individualised care, balancing oncological efficacy with cardiovascular safety to improve patient outcomes.

## 1 Introduction

Prostate cancer (PCa) is a significant global health challenge, ranking fourth in incidence worldwide, with over 1.4 million new cases and nearly 400 000 deaths reported in 2022 (1). Its burden is projected to rise significantly, with the number of cases expected to more than double by 2040 (2). Regionally, PCa is also prevalent. In Singapore, it is the most diagnosed cancer in males, accounting for 17.4% of male cancers from 2018 to 2022 (3). In Malaysia, the lifetime risk varies across ethnic groups: Chinese (1 in 72), Indians (1 in 108), and Malays (1 in 117) (4). The management of PCa-related cardiometabolic risk presents distinct challenges in this region. In Singapore, where PCa is the most prevalent male cancer (3), patients are frequently diagnosed at more advanced stages compared with Western cohorts, and androgen deprivation therapy (ADT) use is correspondingly high. A real-world study of ADT in Asian patients (the READT study, which included Singapore and Malaysian centres) has reported that metabolic complications, including weight gain, hyperglycaemia, and dyslipidaemia, were highly prevalent within the first year of ADT, reinforcing the need for structured early monitoring (5). In Malaysia, considerable heterogeneity in healthcare access across urban and rural settings means that cardiology referral pathways recommended by international guidelines may not be uniformly achievable, necessitating a pragmatic, tiered approach (6). These regional realities informed the development of the present consensus recommendations.

ADT is the standard treatment for advanced PCa, targeting androgen receptor (AR) signalling (7, 8). Pharmacological ADT typically involves the use of gonadotropin-releasing hormone (GnRH) or luteinising hormone-releasing hormone (LHRH) agonist (e.g., leuprolide) or GnRH/LHRH antagonist (e.g., degarelix) while AR-targeted therapies (e.g., enzalutamide, abiraterone) provide alternative suppression pathways (9). Despite their effectiveness, these treatments are associated with cardiometabolic complications, including increased risks of diabetes and cardiovascular disease (CVD) (10).

Recognising the need for region-specific guidance on CVD risk management, a multidisciplinary panel of 12 experts from Malaysia and Singapore developed evidence-based consensus recommendations for assessing, monitoring, and managing cardiometabolic risks in patients receiving ADT/AR-targeted therapies. This paper presents these recommendations, aiming to support clinicians in delivering safer and more holistic care.

## 2 Methods

An expert panel comprising 12 clinicians from Malaysia and Singapore, specialising in PCa and/or CVD management, was convened to develop evidence-based consensus statements. The panel was selected on the basis of a sampling strategy applied across tertiary academic institutions, to capture specialists from both public and private healthcare sectors. Eligibility criteria included >10 years of relevant clinical and/or academic experience; active involvement in related clinical practice, research, or guideline development; and evidence of peer-reviewed publications or recognised leadership in the field of metastatic prostate cancer. The panel comprised specialists from both public and private healthcare sectors and included one cardiologist, two onco-cardiologists, five urologists, two clinical oncologists, one radiation oncologist, and one medical oncologist. A modified Delphi method, prospectively designed with two voting rounds, was applied (11). The panel identified three primary areas of focus: (i) baseline evaluation and screening for risk factors before initiating ADT/AR-targeted therapy; (ii) treatment with ADT/AR-targeted therapy; and (iii) follow-up plans, strategies for preventing cardiometabolic complications, and management of those complications.

A targeted literature search was conducted via PubMed (keywords included abiraterone, androgen deprivation therapy, apalutamide, cardiometabolic, cardiovascular, darolutamide, enzalutamide, gonadotropin releasing hormone, luteinizing hormone-releasing hormone, metabolic, monitoring, prevention, prostate cancer, referral, and risk assessment) to identify high-level evidence (randomised controlled trials, systematic reviews, and meta-analyses) published over the past decade. Articles identified in the bibliographies of the retrieved articles and deemed relevant were also included. Thirteen initial statements were drafted based on the literature. A steering committee (KC, JT, TO) proposed initial draft statements based on a review of current evidence and gaps identified in the literature, with a focus on topics with relevance to the regional context.

At the first meeting, the panel reviewed these draft statements, presented findings from the literature and other relevant sources deemed appropriate, and shared their clinical experience. Statements that did not achieve consensus but demonstrated moderate agreement were reviewed and refined for clarity, including rewording for ambiguity, consolidation of overlapping items, or minor structural modification, while preserving their conceptual intent. Following the meeting, the statements were subjected to an anonymous voting process through a digital platform in which panellists evaluated the practicability of each recommendation. Comments from panellists were systematically reviewed (during the first-round meeting) and used to guide revision of wording and structure where appropriate. Statements that were deemed not applicable or appropriate were omitted following second-round voting.

At the second meeting, the panel discussed, revised, and finalised the consensus statements. A statement was accepted if a predetermined threshold of 75% or more panellists selected either ‘accept completely’ or ‘accept with minor reservation’ from the voting options, which also included ‘accept with major reservation’, ‘reject with reservation’, and ‘reject completely’. This agreement threshold was aligned with the common practices in international Delphi consensus studies (12, 13). Full voting records for all accepted and rejected statements are provided as **Supplementary Material**.

## 3 Recommendations

The panel formed consensus around 12 statements (**Table 1**); one statement was rejected. The rationale for each is outlined below.

**Table 1:** List of accepted consensus statements.

Focus area	No.	Statement
Baseline evaluation and screening for risk factors before initiating ADT/AR-targeted therapy	1	A baseline evaluation of lifestyle risk factors and comorbid conditions is essential for patients with PCa before initiating ADT or AR-targeted therapy. (Agreement: 100%: Accept completely 100%)
	2	A simplified CVD risk assessment checklist should be considered for all patients to categorise their CVD risk before treatment. (Agreement: 100%: Accept completely 66.7%; Accept with minor reservation 33.3%)
Treatment with ADT/AR-targeted therapy	3	The treatment plan for patients on ADT or AR-targeted therapy should include strategies to mitigate the risk of associated comorbidities. (Agreement: 100%: Accept completely 83.3%; Accept with minor reservation 16.7%)
	4	A structured management protocol tailored to CVD risk categories could be implemented to optimise patient care. (Agreement: 100%: Accept completely 83.3%; Accept with minor reservation 16.7%)
	5	For patients with significant pre-existing CVD, GnRH antagonists can be considered over GnRH agonists and surgical castration. (Agreement: 100%: Accept completely 83.3%; Accept with minor reservation 16.7%)
	6	Patients with symptoms or a high CVD risk should be referred for appropriate care. (Agreement: 83.3%: Accept completely 50%; Accept with minor reservation 33.3%; Accept with major reservation 16.7%)
	7	The use of ADT or AR-targeted therapy should consider the cardiovascular risk profile of the patient. (Agreement: 83.3%: Accept completely 83.3%; Accept with major reservation 8.3%; Reject completely 8.3%)
Follow-up plans, strategies for preventing cardiometabolic complications, and management of those complications	8	Cardiometabolic parameters should be monitored after the initiation of ADT or AR-targeted therapy. (Agreement: 100%: Accept completely 75%; Accept with minor reservation 25%)
	9	The frequency of cardiometabolic monitoring should be individualised after the initiation of ADT or AR-targeted therapy. (Agreement: 100%: Accept completely 91.7%; Accept with minor reservation 8.3%)
	10	Counselling on lifestyle modification is recommended during follow-up visits to support CVD risk management. (Agreement: 100%: Accept completely 100%)
	11	The modified ABCDE Cardio-Oncology clinical framework* should be integrated into routine care for patients receiving ADT or AR-targeted therapy to help mitigate CVD risks. (Agreement: 100%: Accept completely 75%; Accept with minor reservation 25%)
	12	An integrated care model involving various specialties to manage cardiometabolic conditions in patients receiving ADT or AR-targeted therapy is preferred. (Agreement: 100%: Accept completely 66.7%; Accept with minor reservation 33.3%)

*The modified ABCDE framework comprises: A, Assess cardiovascular risk; B, Blood pressure; C, Cholesterol and cigarette cessation; D, Diabetes, diet/weight management; and E, Exercise. ADT, androgen deprivation therapy; AR, androgen receptor; CVD, cardiovascular disease; GnRH; gonadotropin-releasing hormone; PCa, prostate cancer.

### 3.1 Focus area 1: baseline evaluation and screening for risk factors before initiating ADT/AR-targeted therapy

#### 3.1.1 Statement 1: a baseline evaluation of lifestyle risk factors and comorbid conditions is essential for patients with PCa before initiating ADT or AR-targeted therapy

Accepted (agreement: 100%)

A baseline evaluation of lifestyle risk factors and comorbid conditions is a critical first step in the management of patients with PCa undergoing ADT/AR-targeted therapy. Lifestyle factors (e.g., smoking, physical inactivity, poor diet) contribute substantially to the development and progression of CVD, increasing the risk of adverse outcomes during cancer treatment. Smoking has been linked to a nearly fivefold increase in the likelihood of coronary heart disease (14) and is also associated with an elevated risk of high-grade PCa (15). Early detection of comorbidities, such as hypertension and diabetes, offers opportunities to optimise a patient’s cardiovascular profile before initiating therapy. To mitigate these risks, and in line with the *Journal of the American College of Cardiology: CardioOncology* consensus, it is recommended to measure and manage key cardiometabolic parameters including blood pressure, lipids, and haemoglobin A1c prior to ADT initiation (16).

#### 3.1.2 Statement 2: a simplified CVD risk assessment checklist should be considered for all patients to categorise their CVD risk before treatment

Accepted (agreement: 100%)

Given the well-established CVD risks associated with ADT and AR-targeted therapies, a streamlined approach to risk stratification is recommended before treatment. A simplified checklist is favoured over complex tools (e.g., American College of Cardiology Atherosclerotic Cardiovascular Disease Risk Calculator) for its ease of use in busy clinical settings. The checklist shown in **Figure 1** categorises patients into high, intermediate, or low CVD risk based on clinical history and key risk factors, including diabetes, smoking, hypercholesterolaemia, and hypertension (17, 18). The original inclusion of novel hormonal agent use was removed in the current adaptation to avoid misclassifying patients as intermediate risk before treatment. To improve the workflow, it is suggested that the checklist be pre-filled or completed by nursing staff, reducing clinician burden and promoting consistent application.

**Figure 1:**
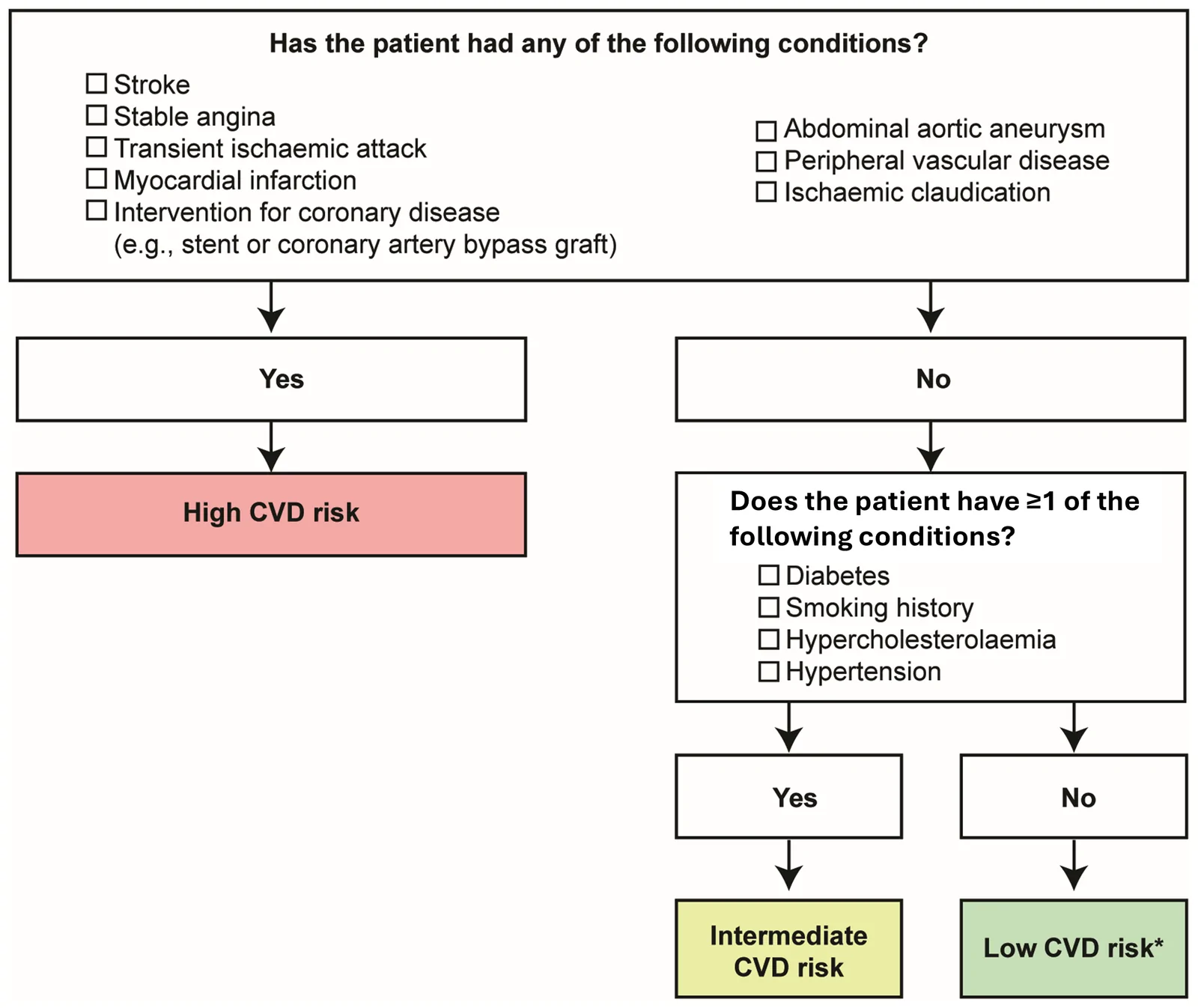
Checklist for CVD risk assessment and stratification. Adapted from: Merseburger AS, Bakshi G, Chen DY, Chiong E, Jabbour M, Joung JY, et al. World J Urol 2024;42:156, which was originally adapted from Davey P, Alexandrou K. Int J Clin Pract 2022:2976811, https://creativecommons.org/licenses/by/4.0/. *A patient’s risk level may transition from ‘Low Risk’ to ‘Intermediate Risk’ or ‘High Risk’ after 2 or 3 years of hormonal plus novel hormonal agent treatment. CVD, cardiovascular disease.

### 3.2 Focus area 2: treatment with ADT/AR-targeted therapy

#### 3.2.1 Statement 3: the treatment plan for patients on ADT or AR-targeted therapy should include strategies to mitigate the risk of associated comorbidities

Accepted (agreement: 100%)

Patients with PCa undergoing ADT/AR-targeted therapies are at an increased risk of developing cardiometabolic comorbidities. In a full cohort study of 1244 PCa patients who received ADT and 1806 non-users, ADT use was associated with a higher heart failure incidence rate (4.00 vs 1.89 per 100 person-years; hazard ratio [HR], 1.72; 95% confidence interval [CI], 1.08–2.73) (19). This association was further supported in a propensity score–matched study (1088 ADT users and 1088 non-users), which found an adjusted HR of 1.92 (95% CI, 1.15–3.18), reinforcing the link between ADT use and increased heart failure risk in patients with PCa (19). Similarly, patients treated with AR-targeted agents such as abiraterone showed a significantly elevated risk of both major composite adverse events (i.e., diabetes, hypertension, congestive heart failure, dysrhythmia, coronary artery disease; HR, 1.77; 95% CI, 1.53–2.05; p<0.001) and minor composite adverse events (HR, 1.24; 95% CI, 1.05–1.47; p=0.01) compared with those not receiving treatment (20). Proactively addressing comorbid conditions through routine monitoring, lifestyle modifications, and pharmacologic interventions can greatly reduce adverse effects and enhance treatment success (**Table 2**) (21–23).

**Table 2:** Strategies to mitigate the complications associated with ADT/AR-targeted therapy.

Complication	Strategy
Cardiometabolic disease	Dietary adjustments (22) Smoking cessation (22) Exercise therapy (22) Monitoring and medical optimisation of cardiovascular risk factors (e.g., hypertension, diabetes, dyslipidaemia) (22)
Bone health	Pharmacologic interventions (e.g., vitamin supplementation, bone resorptive agents [bisphosphonates and monoclonal antibodies] and selective oestrogen receptor modulators) (20)
Mental health (e.g., fatigue, depressive symptoms, sleep disruption, distress, and anxiety)	Behavioural interventions (e.g., cognitive behavioural therapy and physical activity) (20, 21)
Sexual health	Cognitive behavioural therapy (20, 21)

ADT, androgen deprivation therapy; AR, androgen receptor.

#### 3.2.2 Statement 4: a structured management protocol tailored to CVD risk categories could be implemented to optimise patient care

Accepted (agreement: 100%)

A structured management protocol based on CVD risk categories helps ensure timely, appropriate interventions to minimise cardiovascular complications in patients receiving ADT/AR-targeted therapy. As shown in **Figure 2**, risk stratification facilitates appropriate treatment selection, monitoring intensity, and specialist referrals according to CVD risk levels (17, 18). This approach enhances patient safety by aligning the intensity of cardiometabolic management with individual risk profiles, while maintaining oncological efficacy. To ensure practical applicability across diverse healthcare environments, it is recommended that institutions adapt the protocol to their specific clinical settings, patient demographics, and available resources.

**Figure 2:**
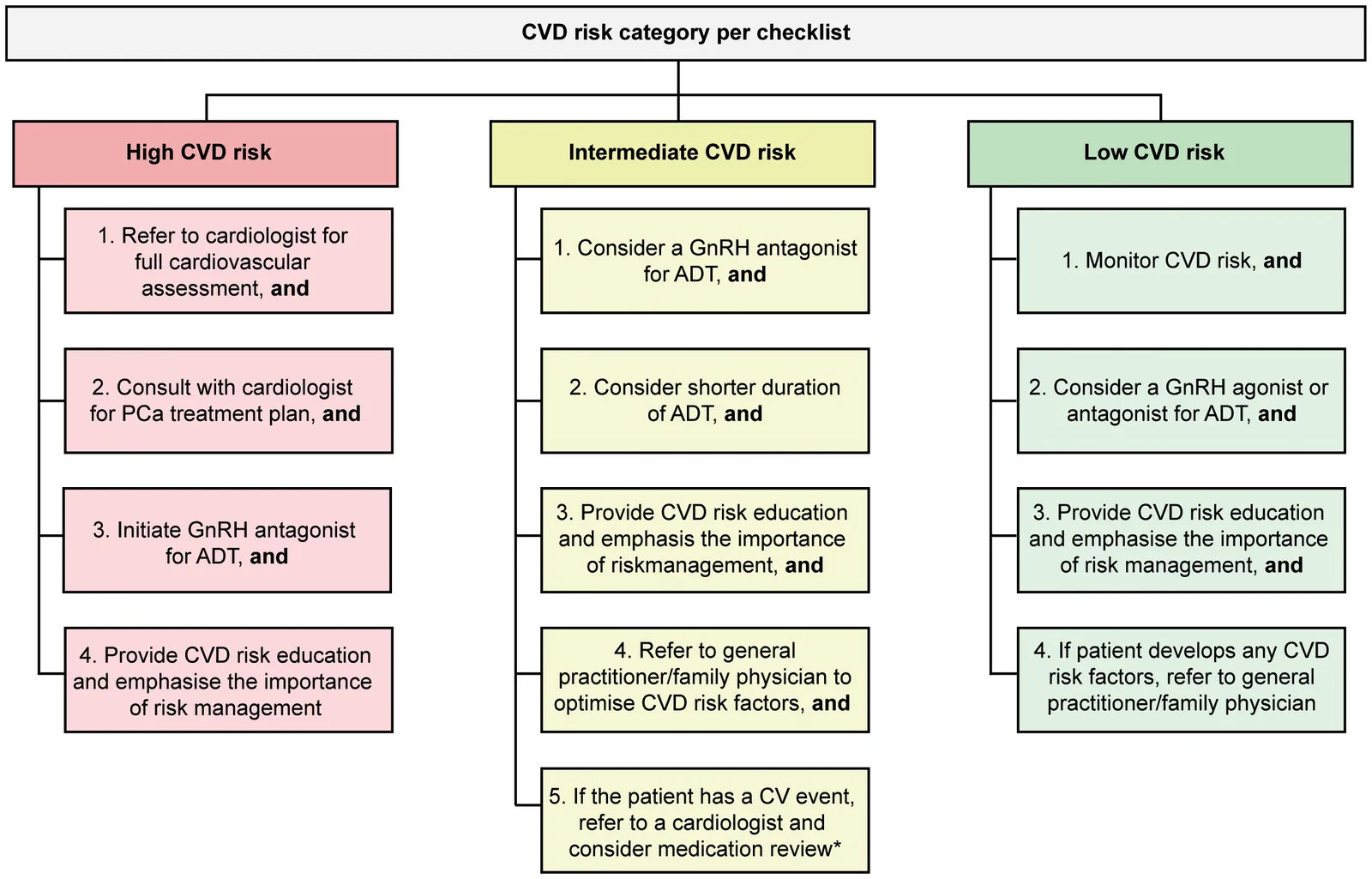
The management steps for minimising cumulative CVD risk at ADT initiation. Source: Merseburger AS, Bakshi G, Chen DY, Chiong E, Jabbour M, Joung JY, et al. World J Urol 2024;42:156, which was originally adapted from an algorithm developed by Davey P, Alexandrou K. Int J Clin Pract 2022:2976811, https://creativecommons.org/licenses/by/4.0/. *Referral to cardiologists is recommended but is subject to each country’s healthcare system and resources. ADT, androgen deprivation therapy; CV, cardiovascular; CVD, cardiovascular disease; GnRH; gonadotropin-releasing hormone; PCa, prostate cancer.

#### 3.2.3 Statement 5: for patients with significant pre-existing CVD, GnRH antagonists can be considered over GnRH agonists and surgical castration

Accepted (agreement: 100%)

Significant pre-existing CVD refers to conditions severe enough to influence treatment decisions, particularly those associated with an elevated risk of major adverse cardiovascular events (MACEs) or those in which certain therapies may exacerbate complications, for example, increasing the risk of transient ischaemic attack (24). Two prospective randomised trials have been reported, designed to identify and compare the cardiac risk associated with PCa medical treatment options. The PRONOUNCE trial (n=545, randomised degarelix vs leuprolide in PCa patients with pre-existing CVD) did not demonstrate a significant difference in major adverse cardiovascular events at 12 months (primary endpoint not met; HR, 1.283, 95% CI, 0.589–2.794), although the trial was limited by early termination and lower-than-expected event rates (25). Similarly, a number of large-scale real-world observational studies have reported no difference or only a slight greater association of GnRH antagonists with cardiovascular risk compared with that of GnRH agonists (26–28). Conversely, the HERO trial (relugolix vs leuprolide, n=934) demonstrated a significant 54% reduction in major adverse cardiovascular events with relugolix (29). Systematic reviews and meta-analyses also suggest that GnRH antagonists are associated with a lower cardiovascular risk than GnRH agonists. In a cohort of PCa patients treated with GnRH antagonist (n=499) or GnRH agonist (n=15 127), GnRH antagonist use was linked to a 33% lower risk of MACEs (adjusted HR, 0.67; 95% CI, 0.46–0.96) and an 84% lower risk of composite cardiovascular events (adjusted HR, 0.16; 95% CI, 0.05–0.50) (30). A pooled analysis also found lower event rates (2.9% vs 4.8%) and reduced odds of all-cause mortality (odds ratio [OR], 0.58; 95% credible interval, 0.32–1.08) in GnRH antagonist users (10). Meta-analyses further support the cardioprotective profile of GnRH antagonists, particularly degarelix, with a significant reduction in MACE incidence (relative risk [RR], 0.59; 95% CI, 0.41–0.84; p=0.003; *I*^2^ = 84%) (RR, 0.60; 95% CI, 0.41–0.88; p=0.008) (31, 32).

The higher cardiac risk observed with GnRH agonists may be attributed to their potential to trigger atherosclerotic plaque instability and rupture, potentially mediated by compensatory increase in follicle-stimulating hormone (33).

Reflecting these findings, the 2022 European Society of Cardiology guidelines recommend considering GnRH antagonists for patients with symptomatic coronary artery disease who require ADT (34). Similarly, at the Advanced Prostate Cancer Consensus Conference (APCCC), 71% of experts favoured the use of GnRH antagonists in patients with advanced PCa and a recent history of major cardiovascular events, compared with only 5% of experts favouring GnRH agonists for such patients (33). Although surgical castration is an effective androgen suppression option, it is infrequently used due to its irreversible nature and the potential psychological burden on patients (35). Overall, the cumulative evidence indicates that GnRH antagonists should be considered the preferred option in patients with substantial cardiovascular risk to mitigate potential treatment-related complications.

#### 3.2.4 Statement 6: patients with symptoms or a high CVD risk should be referred for appropriate care

Accepted (agreement: 83.3%)

Timely referral to appropriate care is crucial for patients with PCa who exhibit symptoms suggestive of CVD (e.g., chest pain, shortness of breath, palpitations) or those identified as having high cardiovascular risk (36). Such symptoms may indicate underlying heart disease or cardiometabolic complications, which can be exacerbated by ADT/AR-targeted agents (36). Referral to appropriate care (e.g., primary care, cardiologists, cardio-oncology clinics) (if they are not on follow-up) ensures that patients receive focused assessment and management tailored to their cardiovascular risks. The evaluation should ideally involve advanced diagnostic tools such as electrocardiogram (ECG), coronary calcium scoring, or the use of cardiac biomarkers. In addition, specialist care facilitates optimisation of cardioprotective therapies (e.g., statins, antihypertensives, antiplatelet agents), structured monitoring protocols, and comprehensive lifestyle interventions to reduce cardiovascular burden (37). Recognising the variability in healthcare infrastructure, particularly in rural or resource-limited areas such as certain regions of Malaysia, the panel recommended allowing flexibility in referral pathways and a broad definition for ‘appropriate care’. For instance, in areas where cardiology services are not readily accessible, it may be appropriate for general physicians or other specialists trained in managing cardiovascular conditions to provide suitable care. This pragmatic approach ensures that patients receive necessary cardiovascular care without causing significant delays in initiating life-prolonging cancer treatment. In most cases, referral should not delay the commencement of ADT.

#### 3.2.5 Statement 7: the use of ADT or AR-targeted therapy should consider the cardiovascular risk profile of the patient

Accepted (agreement: 83.3%)

When prescribing ADT/AR-targeted therapies for PCa, it is essential to consider the patient’s cardiovascular risk profile to minimise treatment-related complications and optimise outcomes, as different agents carry varying degrees of cardiovascular risk (38). Enzalutamide has been associated with an increased risk of acute myocardial infarction, coronary artery disease, and high-grade hypertension, while abiraterone and apalutamide have been linked to a higher incidence of angina pectoris and myocardial infarction (39). In light of these risks, it is equally important to engage patients in discussions about the cardiovascular implications of their treatment options. However, physicians should be mindful of a cultural tendency towards a more paternalistic role of healthcare providers, particularly in Malaysia, where patients may have expectations of delegating the decision-making related to their care (40). Thus, it is especially important to actively promote shared decision-making, empower patients, and foster in patients a sense of accountability for their choices (41). This approach not only enhances patient satisfaction but also ensures that the selected therapy aligns with the patient’s individual risk profile and treatment goals (41, 42).

### 3.3 Focus area 3: follow-up plans, strategies for preventing cardiometabolic complications, and management of those complications

#### 3.3.1 Statement 8: cardiometabolic parameters should be monitored after the initiation of ADT or AR-targeted therapy

Accepted (agreement: 100%)

#### 3.3.2 Statement 9: the frequency of cardiometabolic monitoring should be individualised after the initiation of ADT or AR-targeted therapy

Accepted (agreement: 100%)

Following the initiation of ADT/AR-targeted therapy, regular monitoring of cardiometabolic parameters is essential to detect treatment-related metabolic disturbances and manage cardiovascular risk. ADT has been shown to increase fasting glucose by 4.8%, haemoglobin A1c by 2.7%, triglycerides by up to 20.6%, and body weight by 1.09 kg (5). AR-targeted therapies are also associated with higher risks of hypertension (RR, 1.69), ischaemic heart disease (RR, 1.84), and arrhythmia (RR, 1.38) (43). Regular monitoring of blood pressure, body mass index, haemoglobin A1c or fasting blood sugar, and lipid levels is therefore recommended. The importance of ongoing surveillance was also emphasised in expert discussions at the 2024 APCCC (33). However, a uniform monitoring schedule may not be appropriate for all patients, and there is a lack of evidence from controlled trials on optimal timing. Evidence from an Asian-focused prospective registry study, which included patients from Malaysia and Singapore, indicated that the first year of ADT is a critical period for metabolic changes (5), which may necessitate regular assessments in the first year, conducted at 6 and 12 months to detect early complications (44). Beyond this period, monitoring is recommended every 3 months for high-risk or poorly controlled patients, and every 6–12 months for those with low risk or well-controlled profiles.

#### 3.3.3 Statement 10: counselling on lifestyle modification is recommended during follow-up visits to support CVD risk management

Accepted (agreement: 100%)

#### 3.3.4 Statement 11: the modified ABCDE Cardio-Oncology clinical framework should be integrated into routine care for patients receiving ADT or AR-targeted therapy to help mitigate CVD risks

Accepted (agreement: 100%)

Integrating lifestyle modification counselling into follow-up visits is a fundamental strategy for reducing CVD risk in patients undergoing ADT/AR-targeted therapy for PCa. Evidence shows that 62% (95% CI, 49%–74%) of coronary events can be prevented through adherence to healthy behaviours, including maintaining a healthy body mass index, smoking cessation, regular physical activity, moderate alcohol intake, and a balanced diet (45). The ABCDE framework is a structured, guideline-aligned approach for implementing these interventions in clinical practice (**Table 3**) (46, 47). Supported by real-world evidence, interventions such as smoking cessation (OR, 0.57) and adherence to a healthy lifestyle (OR, 0.52) have been linked to significant reductions in cardiovascular events (48).

**Table 3:** The modified ABCDE framework (40, 41).

Framework	Component
A	Assess risk
B	Blood pressure control (<140/90 mmHg)
C	Cholesterol management and cigarette cessation
D	Diabetes screening, diet, and weight management
E	Exercise

#### 3.3.5 Statement 12: an integrated care model involving various specialties to manage cardiometabolic conditions in patients receiving ADT or AR-targeted therapy is preferred

Accepted (agreement: 100%)

Given the wide-ranging systemic effects of ADT/AR-targeted therapies, a multidisciplinary approach is vital to optimising both oncological and cardiometabolic outcomes. There is some evidence that such outcomes may be improved by integrated care (49), and strong expert consensus endorses collaborative care involving urologists, oncologists, cardiologists, endocrinologists, primary care physicians, and other specialists, to enable comprehensive, coordinated management (28, 50). This is especially important given the heterogeneity of PCa presentations, and the need to balance cancer control with cardiovascular safety (29, 51, 52). A multidisciplinary team can help navigate clinical uncertainties arising from emerging diagnostics and therapies that may fall outside the scope of current guidelines (29). In such cases, cross-specialty input supports nuanced, patient-centred decisions. As the management of PCa continues to advance, the role of multidisciplinary teams will become increasingly vital. Future research should focus on identifying the optimal organisational structures, processes, and success metrics for interdisciplinary care models (29).

### 3.4 Rejected statement

#### 3.4.1 A baseline ECG should be performed in all patients with PCa before initiating ADT or AR-targeted therapy (agreement: 41.7%)

Consensus was not reached regarding this statement recommending a baseline ECG for all PCa patients before initiating ADT/AR-targeted therapy, due to multiple feasibility and clinical concerns. First, there is a lack of strong evidence supporting the utility of ECGs in improving cardiovascular risk stratification in asymptomatic individuals (53). Additionally, in many healthcare settings, particularly in community clinical settings, ECG machines may not be readily available in non-cardiology departments such as urology or oncology, making routine implementation challenging. The added logistical burden of testing, combined with the limited expertise among non-cardiology specialists in interpreting ECGs, could introduce delays or lead to misinterpretation, further complicating patient management without proven evidence of benefits. Moreover, the panel noted that introducing routine ECG screening could potentially detract from the primary objective of initiating timely cancer therapy, especially in the absence of cardiovascular symptoms or high-risk characteristics. As such, while ECGs may be appropriate for selected high-risk or symptomatic patients, the panel did not endorse universal ECG screening prior to ADT initiation.

## 4 Discussion

While these consensus statements offer expert-informed guidance for managing cardiometabolic risk in patients receiving ADT/AR-targeted therapies, limitations should be acknowledged. The convening of a more expansive panel comprising a broader spectrum of healthcare professionals (e.g., primary care physicians, endocrinologists, nurses) can facilitate more comprehensive representation across diverse institutional practices and clinical environments. Future collaborations with wider stakeholder groups may help refine and expand these recommendations. These recommendations were developed based on expert consensus and available evidence, providing practical guidance for clinicians managing cardiometabolic risk in PCa patients. However, as clinical practice evolves, real-world data and long-term outcome studies will be essential to further optimise these recommendations and ensure effective implementation across diverse healthcare environments.

The management of cardiometabolic risks in patients with PCa receiving ADT/AR-targeted therapy requires a structured, multidisciplinary approach. This consensus document highlights the importance of baseline evaluation, individualised risk assessment, proactive monitoring, and lifestyle counselling to minimise treatment-related complications. The adoption of a practical, risk-adapted framework such as the modified ABCDE model can help balance oncological efficacy with cardiovascular safety. While offering standardised guidance, the consensus also supports flexibility for institutions to adapt protocols based on available resources. Ongoing research and real-world data will be key to refining these recommendations and enhancing patient outcomes across diverse clinical settings.
