# Supplementary material for: Management of cardiometabolic risk in prostate cancer: expert consensus from Singapore and Malaysia
Source: Front Oncol. 2026 Oct 21;16:1781791. doi: 10.3389/fonc.2026.1781791 (PMC13732470; doi:10.3389/fonc.2026.1781791)
Supplement: Supplementary file 1 [file DataSheet1.pdf]

Supplementary Material

1 Supplementary Appendix

Full voting records for all accepted and rejected statements.

Focus area 1 – Baseline evaluation and screening for risk factors before initiating androgen deprivation therapy (ADT) or androgen receptor (AR)-targeted therapy

		**Response options* (%)**					**A statement was accepted only if (A + B)% ≥ 75%**		
**Statement #**	**Drafted statements**	**A**	**B**	**C**	**D**	**E**	**A + B (%)**	**Accepted**	**Voted down**
1	A baseline evaluation of lifestyle risk factors and comorbid conditions is essential for patients with prostate cancer (PCa) before initiating ADT or AR-targeted therapy.	100	0	0	0	0	100	✓	
Rejected	A baseline electrocardiogram should be performed in all patients with PCa before initiating ADT or AR-targeted therapy.	17	25	0	17	42	42	X	
2	A simplified cardiovascular disease (CVD) risk assessment checklist should be considered for all patients to categorise their CVD risk before treatment.	67	33	0	0	0	100	✓	
							**Total:**	2	1

*Response options include A: accept completely; B: accept with minor reservation; C: accept with major reservation; D: reject with reservation; and E: reject completely.

Focus area 2 – Treatment with ADT/AR-targeted therapy

		Response options* (%)					A statement was accepted only if (A + B)% ≥ 75%		
**Statement #**	**Drafted statements**	**A**	**B**	**C**	**D**	**E**	**A + B (%)**	**Accepted**	**Voted down**
3	The treatment plan for patients on ADT or AR-targeted therapy should include strategies to mitigate the risk of associated comorbidities.	83	17	0	0	0	100	✓	
4	A structured management protocol tailored to CVD risk categories could be implemented to optimise patient care.	83	17	0	0	0	100	✓	
5	For patients with significant pre-existing CVD, gonadotropin-releasing hormone (GnRH) antagonists can be considered over GnRH agonists and surgical castration.	83	17	0	0	0	100	✓	
6	Patients with symptoms or a high CVD risk should be referred for appropriate care.	50	33	17	0	0	83	✓	
7	The use of ADT or AR-targeted therapy should consider the cardiovascular risk profile of the patient.	83	0	8	0	8	83	✓	
							**Total:**	5	0

*Response options include A: accept completely; B: accept with minor reservation; C: accept with major reservation; D: reject with reservation; and E: reject completely.

Focus area 3 – Follow-up plans, strategies for preventing cardiometabolic complications, and management of those complications

		Response options* (%)					A statement was accepted only if (A + B)% ≥ 75%		
Statement #	Drafted statements	A	B	C	D	E	A + B (%)	Accepted	Voted down
8	Cardiometabolic parameters should be monitored after the initiation of ADT or AR-targeted therapy.	75	25	0	0	0	100	✓	
9	The frequency of cardiometabolic monitoring should be individualised after the initiation of ADT or AR-targeted therapy.	92	8	0	0	0	100	✓	
10	Counselling on lifestyle modification is recommended during follow-up visits to support CVD risk management.	100	0	0	0	0	100	✓	
11	The modified ABCDE Cardio-Oncology clinical framework should be integrated into routine care for patients receiving ADT or AR-targeted therapy to help mitigate CVD risks.	75	25	0	0	0	100	✓	
12	An integrated care model involving various specialties to manage cardiometabolic conditions in patients receiving ADT or AR-targeted therapy is preferred.	67	33	0	0	0	100	✓	
							**Total:**	5	0

*Response options include A: accept completely; B: accept with minor reservation; C: accept with major reservation; D: reject with reservation; and E: reject completely.
